# Influence of Er:YAG laser irradiation on the outcomes of alveolar ridge preservation at the infected molar sites: a randomized controlled trial

**DOI:** 10.1186/s12903-023-02996-y

**Published:** 2023-05-23

**Authors:** Yong Zhou, Fengying Sun, Zhoujing Zhang, Xinxiu Duan, Xianyan Long, Xiang Liu, Duohong Zou, Jiacai He

**Affiliations:** 1 College & Hospital of Stomatology, Key Lab. of Oral Diseases Research of Anhui Province, Anhui Medical University, Hefei, 230032 China; 2 Department of Dental Implantology, College & Hospital of Stomatology, Anhui Medical University, Hefei, 230032 China; 3 Department of Oral Surgery, Shanghai Key Laboratory of Stomatology, School of Medicine, National Clinical Research Center of Stomatology, Ninth People’s Hospital, Shanghai Jiao Tong University, Shanghai, 200011 China; 4 Department of Stomatology, Suzhou Hospital of Anhui Medical University, Suzhou, 234000 China

**Keywords:** Alveolar bone loss, Tooth extraction, Erbium YAG laser

## Abstract

**Background:**

The purpose of this study was to investigate the socket healing outcome after alveolar ridge preservation at infected molar sites using an erbium-doped yttrium aluminium garnet (Er:YAG) laser.

**Methods:**

Eighteen patients who needed molar extraction and exhibited signs of infection were included and allocated into either the laser group or the control group. Er:YAG laser irradiation for degranulation and disinfection was performed with alveolar ridge preservation (ARP) in the laser group. Traditional debridement with a curette was performed in the control group. Two months after ARP, bone tissue samples were harvested at the time of implant placement for histological analysis. Assessment of dimension changes in alveolar bone was conducted by superimposing two cone-beam computed tomography (CBCT) scans taken at baseline and two months after extraction.

**Results:**

Histologically, after two months of healing, Er:YAG laser treatment resulted in more newly formed bone (laser: 17.75 ± 8.75, control: 12.52 ± 4.99, *p* = 0.232). Moreover, greater osteocalcin (OCN) positive expression and lower runt-related transcription factor 2 (RUNX-2) positive expression were detected in the laser group. However, no statistically significant difference was observed between the two groups. The difference in the vertical resorption of the buccal bone plate was statistically significant between groups (laser: -0.31 ± 0.26 mm, control: -0.97 ± 0.32 mm, p < 0.05). Major changes in ridge width were observed at 1 mm below the bone crest. However, the differences between groups were not significant (laser: -0.36 ± 0.31 mm, control: -1.14 ± 1.24 mm, *p* = 0.171).

**Conclusions:**

ARP with Er:YAG laser irradiation seemed to improve bone healing by regulating osteogenesis-related factor expression in the early stage at infected sites.

**Trial registration:**

The trial was registered on the Chinese Clinical Trial Registry Platform (https://www.chictr.org.cn/) (registration number: ChiCTR2300068671; registration date: 27/02/2023).

## Background

Periodontal disease, endodontic treatment failure and trauma are common causes of tooth extraction [1]. Many studies have shown that the width and height of alveolar bone decrease significantly within the first 6 months of natural healing after tooth extraction due to bone resorption and remodelling [2, 3]. Moreover, the healing process is complicated for extraction sockets affected by odontogenic infections [4]. In one study, more than 16 weeks were needed to achieve 50% new bone formation in infected sockets, while in fresh sockets, only eight weeks were needed. Furthermore, at approximately 5% of extraction sites with infection, only connective tissue infiltration was observed rather than bone formation. Periodontitis was the most common aetiology, followed by periodontal-endodontic lesions and endodontic pathology. Age, hypertension and extraction site were reported as impeding factors of erratic healing [5]. Improving bone regeneration at infected extraction sites is still challenging.

Alveolar ridge preservation is a reliable strategy for reducing the resorption of alveolar bone after tooth extraction [6, 7]. When accompanied by infection or granulation tissue in the socket, such as periodontitis, ARP is reported to be effective in preventing ridge shrinkage, provided that degranulation and disinfection are performed [7–9]. The results of a meta-analysis involving ARP at periodontally compromised extraction sites showed that height reduction and bone volume loss were significantly lower in the ARP group. However, the evidence of positive effects is of low certainty [10].

It was reported that Er:YAG laser might improve early new bone formation by regulating the expression of osteogenesis-related factors [11]. E2F targets gene set, Hspa1a, Dmp1 and Sost were all involved in Er:YAG laser irradiated bone area [12]. Low-level Er:YAG laser irradiation directly enhanced Notch signalling and osteocalcin expression in osteoblast-like cells and promoted calcification [13]. However, the exact mechanism of Er:YAG on bone healing still requires further investigation.

Radical debridement is one of the fundamental steps recommended in bone regeneration at infected sites [14]. Usually, a round burr or curette is used to eliminate residual infection on the surface of the bundle bone [15, 16]. Moreover, some authors have suggested that perforation of the bundle bone with a round burr provides access to the bone marrow, improves the bone supply and promotes bone regeneration [17]. Er:YAG lasers are minimally invasive, accurate and highly efficient, and they are widely used in dentistry, including in the treatment of deep caries and peri-implantitis and in surgical resection. Recently, an Er:YAG laser was used in degranulation when performing ARP [18, 19]. However, the Er:YAG laser was applied in combination with other types of lasers [17, 19]. Whether degranulation with an Er:YAG laser at the infected site has a positive effect on bone regeneration is still controversial.

Consequently, this study aimed to investigate the influence of Er:YAG laser debridement during ARP on the quality and quantity of alveolar bone changes at infected extraction sites.

## Methods

### Study design

This study was a randomized controlled clinical trial in a single centre and was performed at the Department of Dental Implantology, College & Hospital of Stomatology, Anhui Medical University (Hefei, China) from December 2020 to December 2021. The primary aim was to investigate the histomorphometry of new bone, and the secondary aim was to assess the dimensional changes of alveolar bone at extraction sites. All procedures and materials were approved by the Medical Ethical Committee of Stomatology Hospital Affiliated with Anhui Medical University (No. T2020011). All the participants agreed to our treatment plan and signed informed consent before the operation. The manuscript was prepared according to CONSORT guidelines, and the clinical trial was registered on the Chinese Clinical Trial Registry Platform.

The sample size was calculated for alveolar ridge reduction as reported by a meta-analysis of alveolar ridge preservation, that is, 3.5 mm resorption in height in the control group and 2 mm in the ARP group. The standard deviation between the two groups was 1.08 mm [9]. The significance level was set as 0.05, and the beta error was a power of 80%. It was estimated that a total of 8 cases per group were required at a 1:1 ratio. Assuming a dropout rate of 15%, the final calculated sample size was approximately 18 participants, that is, 9 subjects in each group.

### Participants

Patients requiring extraction of maxillary or mandibular molars were recruited. CBCT (NewTom VG, NewTom, Italy) images (voxel size, 0.125 mm; field, 12 × 8 cm; exposure, 5.4 s; energy, 3.6 mA) were taken at baseline. A comprehensive examination was performed to determine whether the following criteria were met.

Inclusion criteria:

1. Molars with periodontitis, periodontal–endodontic lesions, or endodontic pathology without fistula that could not be preserved.
2. Only one extraction bordered by teeth medially and distally.
3. Four intact bony walls with at least 5 mm in height remaining.
4. Implant treatment planned after tooth extraction.
5. No serious systemic disease, such as severe cardiovascular disease or uncontrolled diabetes.
6. Sufficient plaque control and healthy periodontal maintenance (bleeding on probing < 20%, plaque index < 20%).

Exclusion criteria:

1. Patients under the age of 18 years or older than 70 years.
2. Pregnant or lactating women.
3. Patients with acute infection.
4. Patients smoking more than 10 cigarettes/day.
5. Patients taking drugs affecting bone metabolism, such as bisphosphonates, corticosteroids, or nonsteroidal anti-inflammatory drugs, for a long time.
6. In patients with multiple eligible sites, only one tooth site was selected.

Once patients were recruited, randomized assignment of treatment modalities was performed by a computer. The allocation information for each participant was placed into an opaque sealed envelope, which was opened by an operator who was not involved in the experiment just after the extraction. The patients were randomly allocated to the laser group or the control group. All patients were instructed to attend 4 visits over a 2-month study period.

### Alveolar Ridge Preservation

**Fig. 1:**
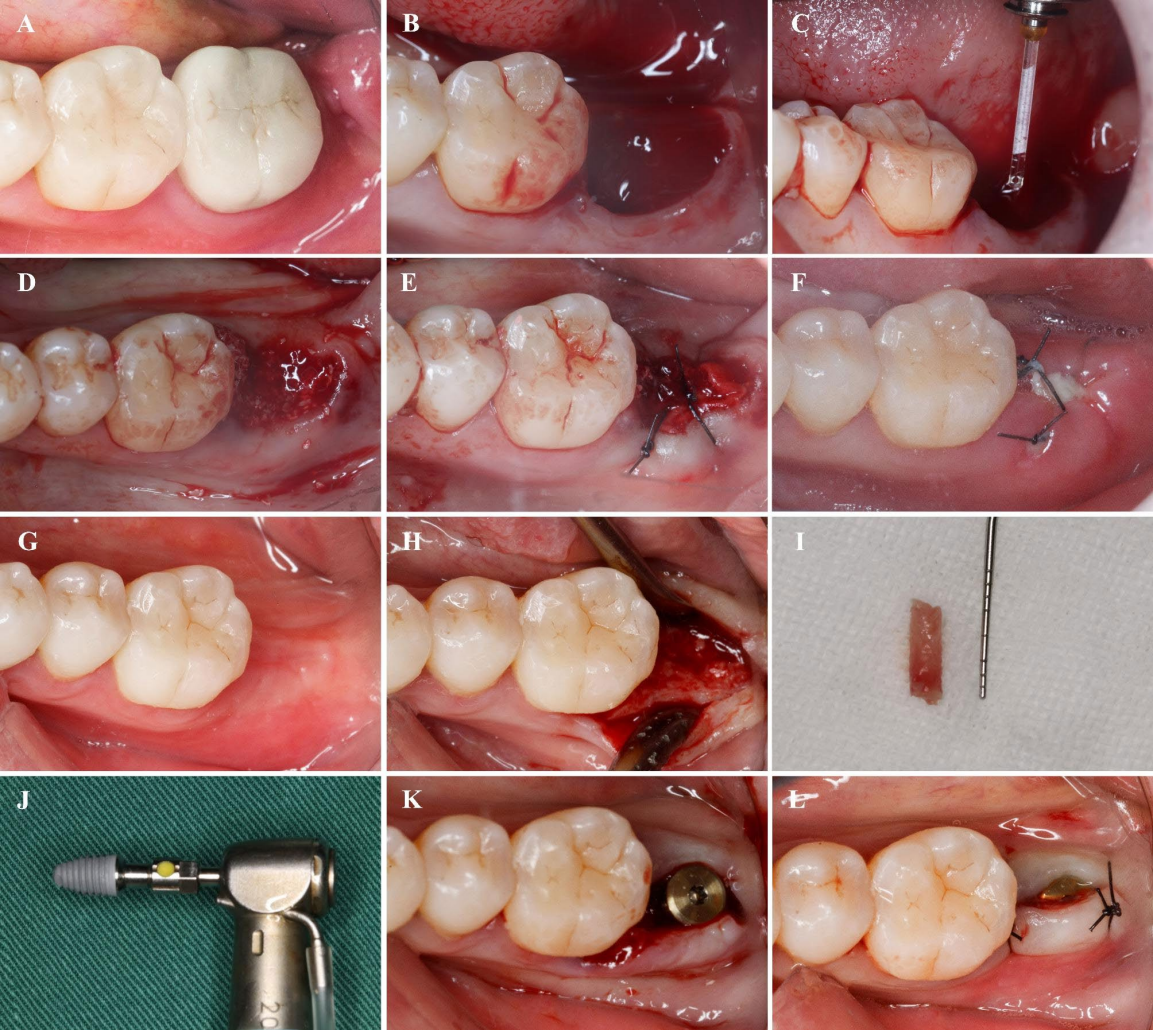
Surgical procedure of ridge preservation in laser group. (**A**) Hopeless mandibular second molar before extraction. (**B**) Socket after tooth extraction. (**C**) Degranulation with Er:YAG laser. (**D**) Bone grafting. (**E**) Wound closure. (**F**) One week after the extraction. (**G**) Two months after the extraction. (**H**) Flapping. (**I**) Bone core harvested. (**J**)Implant. (**K**) Implant and healing abutment placement. (**L**) Wound closure

**Fig. 2:**
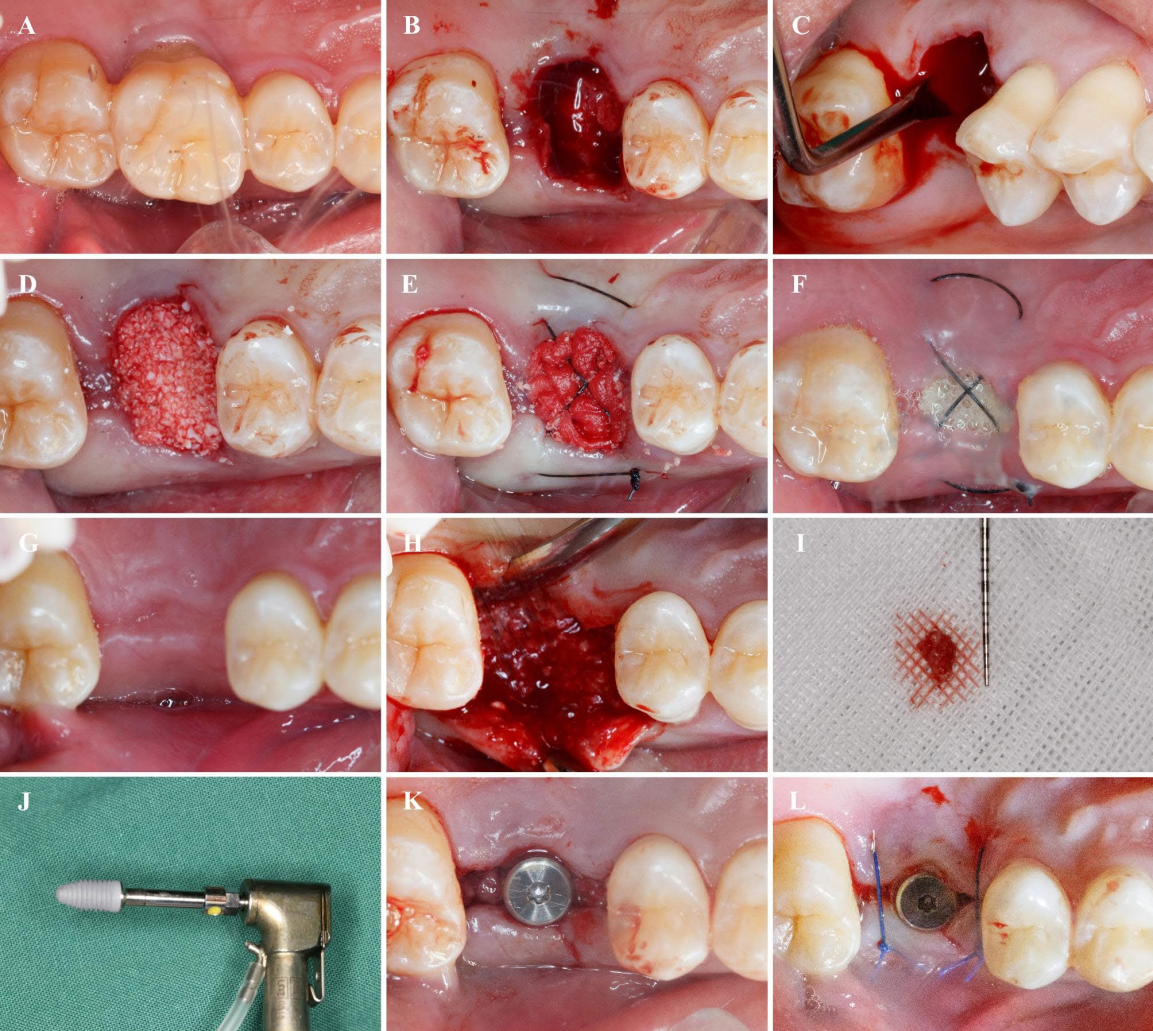
Surgical procedure of ridge preservation in control group. (**A**) Hopeless maxillary first molar before extraction. (**B**) Socket after tooth extraction. (**C**) Degranulation with curette. (**D**) Bone grafting. (**E**) Wound closure. (**F**) One week after the extraction. (**G**)Two months after the extraction. (**H**) Flapping. (**I**) Bone core harvested. (**J**)Implant. (**K**) Implant and healing abutment placement. (**L**) Wound closure

A 0.12% chlorhexidine rinse (Chenpai, Jiangsu, China) was administered, and local anaesthesia with primacaine (4% articaine hydrochloride with 1:100000 adrenaline) was performed. Minimally invasive extraction of the affected tooth was introduced without flapping. The tooth was sectioned when necessary. Care was taken to preserve the integrity of the socket bone and gingival tissue. Patients were excluded if bone fracture occurred during extraction. After extraction, subjects were allocated to receive either laser treatment (laser group) or conventional treatment (control group) according to the randomized method described above. In the laser group, an Er:YAG laser (Lightwalker, Fotona, Slovenia) was used to completely remove the granulation tissue in the extraction socket (Fig. 1). A curette was used to probe the extraction socket gently to ensure that the granulation tissue was completely debrided. The working parameters of the Er:YAG laser were 100 mJ, 20 Hz, water 4, air 2, SP pulse duration, HC14 handpiece, and cylindrical tip with 1.3 mm diameter. In the control group, traditional degranulation with a curette or round burr was performed (Fig. 2). The alveolar socket was rinsed with 0.2% chlorhexidine solution followed by immediate implantation of a bone graft (Bio-OSS, Geistlich Pharma, Wolhausen, Switzerland) in combination with a collagen membrane (Bio-OSS, Geistlich Pharma, Wolhausen, Switzerland) in both groups. Mucosal flaps were closed without coronal advancement using cross-mattress sutures.

All subjects received detailed postoperative oral and written instructions; antibiotic drugs were administered as follows: cefradine 500 mg every 6 h for 3 days (or roxithromycin 300 mg every 24 h for 3 days for patients with penicillin allergy) and tinidazole 500 mg every 12 h for 3 days. Analgesics such as ibuprofen (300 mg every 12 h) were administered if necessary. All patients were given oral hygiene instructions and mouthwash (0.12% compound chlorhexidine solution, 3 times a day for 7 days). One week after surgery, the sutures were removed.

### Implant placement and bone biopsy harvest

CBCT (NewTom VG, NewTom, Italy) images (voxel size, 0.125 mm; field, 12 × 8 cm; exposure, 5.4 s; energy, 3.6 mA) were taken 2 months after ARP to evaluate bone quantity and plan the treatment modality. Under local anaesthesia, full-thickness flaps were elevated. A trephine (Chuangying, Jiangsu, China) core of 3 × 10 mm was obtained with saline irrigation from the preserved area. If the available bone height was lower than 10 mm, the bone sample of maximum length was harvested according to CBCT. Osteotomy was prepared, followed by implant placement. The flaps were repositioned, and the wound was sutured. Patients were given the same postoperative instructions as in the previous surgical procedure. The harvested bone cores were fixed in 10% formalin for histological evaluation.

### Dimensional changes

**Fig. 3:**
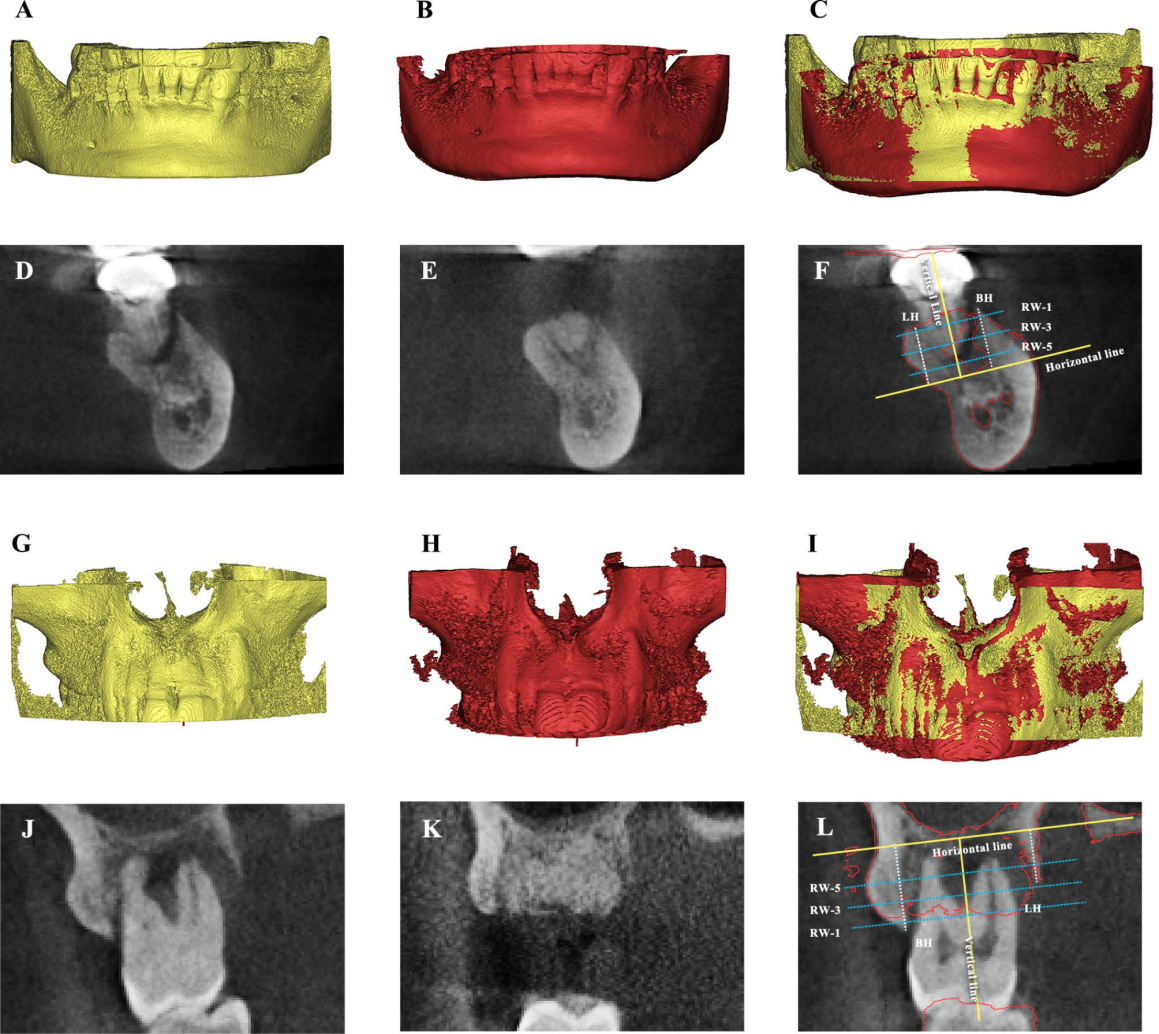
Dimensional changes evaluation of laser group (**A-F**) and control group (**G-L**) with CBCT. (**A, G**) 3D reconstruction of CBCT at baseline. (**B, H**) 3D reconstruction of CBCT two months after ARP. (**B, F**) CBCT at baseline. (**C, I**) Superimposition of two CBCTs at baseline and two months after ARP with anatomical landmarks. (**D, J**) Coronal aspect of CBCT at baseline. (**E, K**) Coronal aspect of CBCT two month after ARP. (**F, L**) Radiographic evaluation of dimensional changes. Vertical measurements: buccal bone (BH), and lingual height (LH). Horizontal measurements: ridge width (RW) at 1 mm (RW-1), 3 mm (RW-3), and 5 mm (RW-5) from the bone crest. (Red line, contour of the alveolar bone two month after ARP)

To perform CBCT measurements, the original DICOM data captured two months after healing were processed, exported to STL format files and superimposed and aligned with the CBCT data at baseline with the 3D medical image segmentation software Mimics (Materialise, Leuven, Belgium). The accuracy of alignment was ensured by a manual check. The process was conducted by an examiner who was uninformed of the treatment allocation. Data analysis was performed according to previously published studies [20, 21]. Two lines perpendicular to each other and intersecting at the apex of the socket were drawn. The line along the central part of the extraction socket was named the vertical reference line, and the other line was named the horizontal reference line. To determine the buccal plate height (BH) and the lingual plate height (LH), another two vertical lines perpendicular to the horizontal reference line crossing the tip of the buccal plate and lingual plate, respectively, were produced. Three lines parallel to the horizontal line were placed at 1 mm, 3 mm, and 5 mm below the crest of the alveolar bone. The buccolingual/palatal width of the alveolar ridge at each of the three lines (RW-1, RW-3 and RW-5) was calculated according to the distance between the two intersection points of the parallel lines with the lateral wall of the buccal bone and lingual/palatal bone. The thickness of the buccal bone plate (BT) was also measured at the same level as the RW at baseline (Fig. 3).

### Histological analysis

The specimens were decalcified and cut medially along the long axis of the bone core. After paraffin embedding, 3 × 10 mm sections were produced medially from the aspect of the tissue surface. Haematoxylin and eosin staining was performed. Immunohistochemical analysis of antibodies against OCN and RUNX2 was performed. For sections involving the bone tissue located under the socket, only the upper part was defined as the ARP area for data analysis.

The relative percentage of new bone (NB) formation, nonmineralized connective tissue (NMCT) area, remnant material (RM) area, OCN-positive expression area and RUNX-2-positive expression area were calculated and measured with ImageJ. The assessor was blinded to the experimental assignment and not involved in the investigation.

### Statistical analysis

Data are presented as the mean ± SD. Data were first tested by Shapiro–Wilk tests to determine if they were normally distributed. Two independent sample t tests were used for intergroup comparisons of patient age, time for socket recovery, dimension changes and histological results. Intragroup comparisons of dimension changes in alveolar bone 2 months after the treatment were determined using the paired t test. Sex, location and smoking habit were evaluated with Fisher’s exact test. The level of statistical significance was set at *p* < 0.05.

## Results

From December 2020 to December 2021, a total of 20 patients were recruited. After clinical examination, two patients did not meet the criteria. After ARP, one patient in the laser group refused treatment because of a change to a workplace far away from our hospital. Three patients dropped out due to COVID-19 and withdrawal of consent (2 in the control group and 1 in the laser group). Data from these patients were not included in the study analysis; thus, 14 patients, 7 in each group, completed the study and were included in the statistical analyses for the primary and secondary outcomes. All patient characteristics are summarized in Table 1. No specific adverse events were recorded, such as persistent swelling and pain, bleeding, nerve injury or infection.

**Table 1:** Baseline characteristics of patients

	Laser	Control	Total	*p* value
**Age (years)**	37 ± 14.04	36.71 ± 12.27	39.21 ± 14.12	0.874
**Gender**				
Male	3	4	7	1.000
Female	4	3	7	
**Location**				
Mandible	5	5	10	0.720
Maxilla	2	2	4	
**Smoking habit**				
No	7	7	12	-
Yes	0	0	0	
**Aetiology**				
periodontitis	1	2	3	0.633
periodontal-endodontic lesion	5	3	8	
endontitis	1	2	3	
**Time for socket recovery(days)**	70.71 ± 14.40	76.57 ± 12,27		0.429

### Dimension changes

At baseline, there was no significant difference between the groups in vertical height or horizontal width at any position (Table 2).

**Table 2:** Vertical and horizontal dimension changes of alveolar ridge (mean ± SD)

		Laser(mm) (N = 7)	Control(mm) (N = 7)	Laser VS Control *p* value
**BH**	**Baseline**	8.61 ± 2.46	7.30 ± 2.60	0.391
	**2 Months**	8.30 ± 2.47	6.33 ± 2.64	
	**Difference**	-0.31 ± 0.26	-0.97 ± 0.32	0.003^*^
	***p*** **value**	0.016^*^	<0.01^*^	
**LH**	**Baseline**	7.49 ± 2.83	6.71 ± 1.88	0.587
	**2 Months**	7.26 ± 2.74	5.98 ± 2.34	
	**Difference**	-0.23 ± 0.20	-0.73 ± 1.08	0.290
	***p*** **value**	0.02^*^	0.079	
**RW1**	**Baseline**	11.12 ± 2.52	13.47 ± 1.94	0.101
	**2 Months**	10.79 ± 2.44	12.33 ± 2.88	
	**Difference**	-0.36 ± 0.31	-1.14 ± 1.24	0.171
	***p*** **value**	0.033^*^	0.038^*^	
**RW3**	**Baseline**	13.94 ± 2.24	15.63 ± 2.20	0.217
	**2 Months**	13.92 ± 2.41	14.90 ± 2.32	
	**Difference**	-0.05 ± 0.34	-0.73 ± 0.41	0.011^*^
	***p*** **value**	0.438	0.004^*^	
**RW5**	**Baseline**	14.73 ± 2.38	16.99 ± 2.16	0.115
	**2 Months**	14.60 ± 2.41	16.73 ± 2.52	
	**Difference**	-0.13 ± 0.094	-0.27 ± 0.55	0.549
	***p*** **value**	0.103	0.145	
**BP-1**	**Baseline**	1.33 ± 0.33	1.11 ± 0.22	0.209
**BP-3**	**Baseline**	1.63 ± 0.60	1.35 ± 0.49	0.399
**BP-5**	**Baseline**	2.06 ± 1.02	1.89 ± 0.86	0.754

Two months after ARP, vertical reduction of alveolar bone occurred buccally and lingually in both groups. The vertical dimension change in the buccal bone plate was more significant. Moreover, the mean reduction of the buccal bone plate in the laser group (0.31 ± 0.26 mm.) was less than that in the control group (0.97 ± 0.32 mm), with a statistically significant difference.

Horizontally, at all measured levels (RW-1, RW-3, and RW-5), loss in the alveolar ridge was detected in both groups (Table 2). Pronounced resorption was detected in the coronal aspects of the ridge (RW-1) in both groups (laser group: -0.36 ± 0.31 mm, control group: -1.14 ± 1.24 mm). At RW-3, the width of the alveolar ridge was only significantly decreased in the control group. However, there were no significant differences in horizontal change at any level between the two groups (p > 0.05).

Similar resorption tendency of the ridge was obtained in mandible and maxilla (Table 3). Significant difference could only be observed when considering the resorption of buccal bone between both groups in mandible.

**Table 3:** Dimension change on CBCT measurements of maxillary and mandible extraction sockets (mean ± SD)

	BH			LH			RW-1			RW-3			RW-5		
	Laser	Control	*p*	Laser	Control	*p*	Laser	Control	*p*	Laser	Control	*p*	Laser	Control	*p*
**Mandible (N = 5 for each group)**															
**Baseline**	9.59 ± 2.45	8.07 ± 2.09	0.384	8.83 ± 2.39	7.13 ± 1.85	0.302	9.64 ± 1.14	10.96 ± 2.68	0.398	12.73 ± 1.35	14.03 ± 2.93	0.449	13.53 ± 1.71	14.25 ± 2.97	0.685
**2 months**	9.18 ± 2.60	7.49 ± 2.44		8.57 ± 2.28	6.58 ± 2.72		9.38 ± 1.03	11.51 ± 3.21		12.65 ± 1.31	14.44 ± 2.84		13.39 ± 1.76	15.83 ± 2.44	
**Changes**	-0.41 ± 0.27	-0.96 ± 0.12	0.02	-0.26 ± 0.24	-1.05 ± 1.24	0.261	-0.26 ± 0.27	-1.28 ± 1.55	0.281	-0.08 ± 0.10	-0.72 ± 0.52	0.051	-0.14 ± 0.12	-0.34 ± 0.69	0.593
**Maxilla (N = 2 for each group)**															
**Baseline**	6.66 ± 0.93	8.75 ± 4.11	-	4.80 ± 0.99	7.52 ± 5.21	-	14.10 ± 1.10	10.45 ± 2.82	-	16.37 ± 1.41	13.69 ± 1.75	-	17.14 ± 1.40	15.31 ± 1.05	-
**2 months**	6.54 ± 0.98	4.02 ± 0.97	-	4.63 ± 1.13	4.78 ± 0.91	-	13.61 ± 1.65	13.97 ± 1.64	-	16.45 ± 2.15	15.83 ± 0.41	-	17.04 ± 1.40	18.51 ± 2.11	-
**Changes**	-0.12 ± 0.04	-0.99 ± 0.69	-	-0.17 ± 0.13	-0.10 ± 0.07	-	-0.49 ± 0.56	-0.85 ± 0.49	-	-0.08 ± 0.74	-0.75 ± 0.18	-	-0.10 ± 0.00	-0.13 ± 0.04	-

### Histological outcomes

#### HE staining

**Fig. 4:**
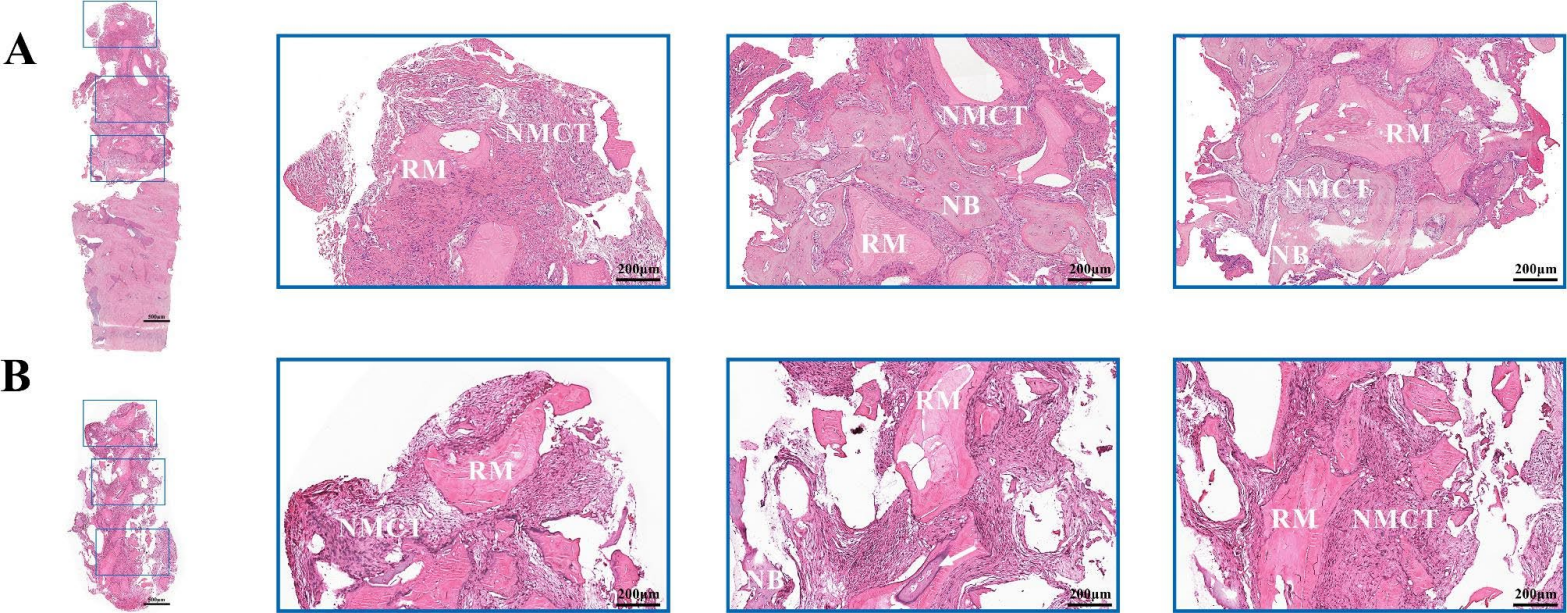
Histomorphological analysis of bone sample with Hematoxylin-Eosin staining (**A**) laser group. (**B**) control group. Blue box, presentation of the upper, middle and lower part of the bone sample at a magnification of 50x. material remnant (RM), non-mineralized connective tissue (NMCT) and new bone (NB).

As shown in Fig. 4, new bone formation was only detected in the lower part of the tissue in both groups. At the top of the tissue, material remnants surrounded by multiple connective tissues were observed. In the laser group, some of the new bone adhered closely to the material remnants, and most of the bone tissue was surrounded by connective tissue. In the control group, the material was surrounded mainly by connective tissue, and there was little new bone formation at the periphery of the connective tissue.

For mandibular extraction sockets, more NB (18.75 ± 5.18) and less RM (36.86 ± 3.77) was observed when Er:YAG laser was involved. However, no significant difference was found for either of these parameters between groups (p > 0.05). For maxillary sockets, the results of NB and RM between the two groups were very close in terms of values except for NMCT. (Table 4)

**Table 4:** Histomorphometric evaluation of bone tissue samples. NMCT, non-mineralized connective tissue, NB, new bone and RM, remnant material area

		NB (%)	RM (%)	NMCT (%)	OCN (%)	RUNX2 (%)
**Mandible**	**Laser** **(N = 5)**	18.75 ± 5.18	36.86 ± 3.77	35.36 ± 7.74	6.06 ± 4.63	2.64 ± 2.19
	**Control**	12.20 ± 4.88	46.11 ± 6.80	36.21 ± 6.88	3.55 ± 3.40	6.30 ± 5.50
	**(N = 5)**					
	***p*** **value**	0.115	0.055	0.874	0.416	0.263
**Maxilla**	**Laser** **(N = 2)**	12.77 ± 0.12	37.83 ± 3.20	34.59 ± 17.88	9.32 ± 8.00	1.96 ± 1.29
	**Control** **(N = 2)**	13.18 ± 8.61	34.65 ± 3.54	52.18 ± 5.07	3.45 ± 3.66	3.78 ± 3.98
	***p*** **value**	-	-	-	-	-
**In total**	**Laser** **(N = 7)**	16.09 ± 3.86	35.52 ± 3.79	33.59 ± 9.39	7.14 ± 5.34	2.41 ± 1.83
	**Control** **(N = 7)**	12.52 ± 5.42	42.29 ± 8.08	39.86 ± 6.28	3.51 ± 3.10	5.45 ± 4.80
	***p*** **value**	0.219	0.093	0.204	0.18	0.177

When performing the analysis regardless of the location, the percentage of NB was greater when Er:YAG laser degranulation was used. As shown in Table 4, the overall NB was 17.75 ± 8.75 in the laser group and 12.52 ± 4.99 in the control group. Moreover, slightly more material remnants were detected in the control group (42.29 ± 8.08) than in the laser group (35.52 ± 3.79). No significant difference was found for either of these parameters between groups (p > 0.05) (Table 4).

### Immunohistochemical analysis

**Fig. 5:**
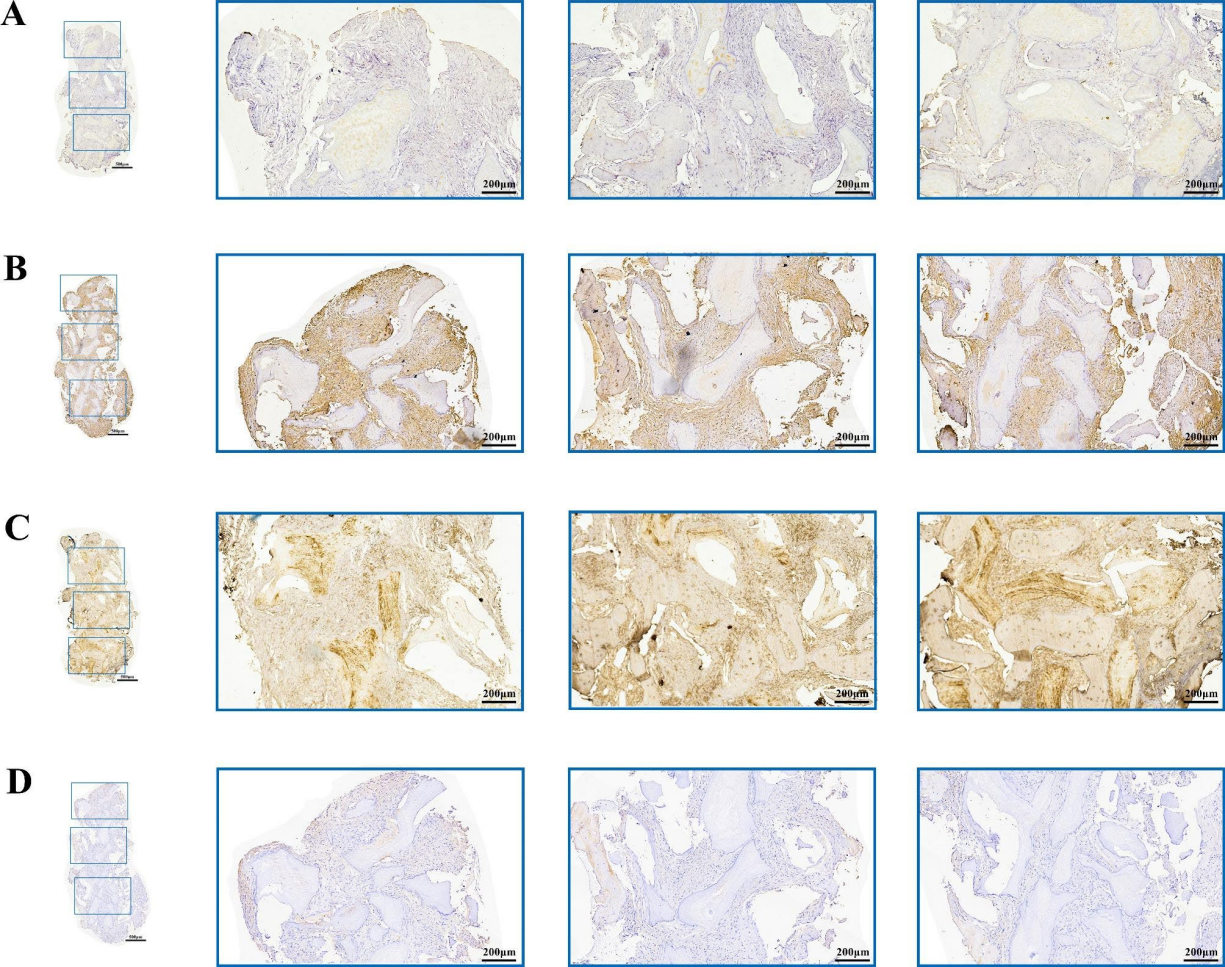
Immunohistochemistry analysis result. (**A**) The detection of RUNX-2 expression in laser group. (**B**) The detection of RUNX-2 expression in control group. (**C**) The detection of OCN expression in laser group. (**D**) The detection of OCN expression in control group. Blue box, presentation of the upper, middle and lower part of the bone sample at a magnification of 50x

Runx-2 was mostly expressed in the nonmineralized connective tissue. A scattered distribution of light-yellow staining was observed in the laser group (Fig. 5A). In the control group, a large area of dark brown staining was detected (Fig. 5B). However, OCN expression exhibited the opposite tendency; there was more positive staining area expressed in the laser group (Fig. 5C and D).

The immunohistological analysis of OCN is shown in Table 4. An increase in OCN expression and decrease in RUNX-2 expression could be obtained in both mandible and maxillary sockets. However, the results did not show a statistically significant difference.

## Discussion

Alveolar ridge preservation is a successful method to maintain the alveolar process after tooth extraction and is also recommended in extraction sites with infection [7, 9]. Er:YAG laser is one of the commonly used laser types in dentistry [22–25]. Our study shows that degranulation and disinfection with an Er:YAG laser can be effective in treating infected tooth extraction sites and that, compared with curette, Er:YAG laser irradiation when performing ARP in infected sites could improve bone regeneration in the early stage.

Most studies involving ARP in infected areas have been based on results at later time points after extraction [26]. There have been few histological studies on early bone tissue healing after extraction site preservation, and the graft materials used in these studies varied. Heberer et al. reported the histomorphometric results of extraction socket healing 6 weeks after ARP with Bio-Oss Collagen. The bone core harvested consisted of 28% new bone, 11% Bio-Oss remnants and 54% connective tissues [27]. Lauren A. Brownfield et al. explored the healing of extraction sockets by histological examination at 2.5 months to 3 months after extraction site preservation and found 37.4% new bone formation in the extraction site preservation group. Because allografts have a rapid resorption rate, only 4.5% of material was detected histologically [28]. In another study exploring healing two months after extraction site preservation, PRF was grafted into the socket. New bone formation was approximately 24.9 ± 3.7% in the socket without filling and 28.1 ± 3.7% when PRF was implanted [29]. In this study, bone formation was less than that reported in the literature above. This might be attributed to the fact that extraction sockets in this study were affected with severe periodontitis or periodontal-endodontic pathology. Moreover, based on the histological findings, a preliminary conclusion can be drawn that bone regeneration was improved after ARP with Er:YAG laser debridement, especially in mandible. However, no significant difference was detected between the groups regarding the parameters investigated above. The histological results need to be interpreted with caution because the tissue sample obtained was small and may not be fully representative of regional healing.

In recent years, the photobiomodulation of the Er:YAG laser has also gained attention; Lin T et al. have shown that Er:YAG laser irradiation promotes gingival soft tissue wound healing and periodontal stem cell proliferation [30]. Interestingly, Er:YAG laser irradiation can also promote bone tissue healing. Ohsugi Y demonstrated using an animal model that the Er:YAG laser irradiation promoted ALP and OCN expression [11]. Zeitouni J et al. found that the trabecular structures at the cut edge were preserved without significant thermal damage or carbonization when using an Er:YAG laser [31]. In a study by Lubart et al., Er:YAG laser irradiation dissociated water molecules, releasing dissociated OH ions that stimulated cell biochemical properties. However, it remains unclear how Er:YAG laser irradiation exerts its biomodulatory effect. In this study, the expression of RUNX-2 and OCN was assessed. Both factors are important osteogenic-related growth factors. RUNX-2 is a transcription factor that improves osteoblastic differentiation and bone formation at an early stage. Moreover, it is a promoter of several osteogenic genes, including OCN. OCN is mostly synthesized and secreted at the mid to late stages of osteogenic differentiation in bone [32, 33]. The detection of RUNX-2 and OCN expression could indicate the stage of mineralization that has been achieved. Greater OCN expression and lower RUNX-2 expression were found in the laser group in this study, although no significant difference was found, which might suggest that the tissue in the laser group was more mature and had healed faster.

For the dimension changes, a statistically significant reduction in labial bone height resorption was observed when the Er:YAG laser was used. It was reported that the thickness of the bone plate plays an important role in bone dimensional changes. Buccal bones thinner than 1.5 mm lose more horizontally compared to thicker ones. In this study, the difference in buccal plate width was not statistically significant at baseline. The benefit of Er:YAG irradiation on buccal bone preservation might be partly attributed to less mechanical stimuli during degranulation with the Er:YAG laser [34]. However, further study is still needed to confirm this hypothesis.

Although successful results were achieved by using an Er:YAG laser for alveolar preservation, the best parameters are controversial. There is no consensus in the literature on the optimal dose, frequency, and intensity parameters. The recommended power settings for the Er:YAG laser are 1–3 W for soft tissue and 1.5-3 W for bone [35]. However, this recommendation was made for cutting tissues. When performing ARP, infected sockets require not only thorough debridement and decontamination but also improvement of bone regeneration by the biomodulatory effect of Er:YAG laser irradiation. Therefore, the best parameters of the Er:YAG laser should be investigated further. According to Lin et al., there is a difference in inducing optimal cell proliferation when applying an Er:YAG laser with varied energy. Yoichi Taniguchi et al. used panel parameters of 70 mJ and 20 Hz for debridement in periodontal defects [23]. Aleksandra Križaj Dumic et al. performed degranulation with an Er:YAG laser at 160 mJ and 15 Hz [18]. Crippa R et al. recommended a power of 2 W and 15 Hz for debridement and decontamination [19]. In this study, the parameters were an energy of 100 mJ, a frequency of 20 Hz and an output power of 2 W. Further experiments should be performed to determine the appropriate laser parameters for ARP.

There are some limitations in this study. First, the bone graft was implanted into the socket. Therefore, the effect of the Er:YAG laser might be influenced by the immunoregulatory response induced by the graft materials. Second, the infected teeth involved in this study were not grouped by aetiology. There is a difference in infection modality between periodontitis and apical periodontitis. Third, the sample size was small. RCTs with larger sample sizes that consider the aetiology of infection and perform ARP without bone grafts are needed to further illustrate the effect of the Er:YAG laser on healing in extraction sockets with infection.

## Conclusions

Within the limitations of the present study, it can be concluded that Er:YAG laser irradiation for degranulation and disinfection is effective and promising compared to curettage treatment. Moreover, the addition of an Er:YAG laser to the postextraction protocol may be especially beneficial for accelerating early bone regeneration.

## Data Availability

The datasets used and/or analyzed during the current study are available from the corresponding author on reasonable request.

## List of abbreviations

Er:YAG: Erbium-doped yttrium aluminium garnet
ARP: Alveolar ridge preservation
CBCT: Cone-beam computed tomography
RUNX-2: Runt-related transcription factor 2
OCN: Osteocalcin
BH: Buccal plate height
LH: Lingual plate height
RW: Buccolingual width of alveolar ridge
BT: Buccal bone plate
NB: New bone formation
NMCT: Nonmineralized connective tissue
RM: Remnant material
